# Persistence of Health Inequalities in Childhood Injury in the UK; A Population-Based Cohort Study of Children under 5

**DOI:** 10.1371/journal.pone.0111631

**Published:** 2014-10-27

**Authors:** Elizabeth Orton, Denise Kendrick, Joe West, Laila J. Tata

**Affiliations:** 1 Lecturer and Specialty Registrar in Public Health, Division of Primary Care, University Park, University of Nottingham, Nottingham United Kingdom; 2 Professor of Primary Care Research, Division of Primary Care, University Park, University of Nottingham, Nottingham United Kingdom; 3 Clinical Associate Professor and Reader in Epidemiology; Consultant Gastroenterologist, Division of Epidemiology and Public Health, Nottingham City Hospital, University of Nottingham, Nottingham, United Kingdom; 4 Associate Professor in Epidemiology, Division of Epidemiology and Public Health, Nottingham City Hospital, University of Nottingham, Nottingham, United Kingdom; UCL Institute of Child Health, University College London, United Kingdom

## Abstract

**Background:**

Injury is a significant cause of childhood death and can result in substantial long-term disability. Injuries are more common in children from socio-economically deprived families, contributing to health inequalities between the most and least affluent. However, little is known about how the relationship between injuries and deprivation has changed over time in the UK.

**Methods:**

We conducted a cohort study of all children under 5 registered in one of 495 UK general practices that contributed medical data to The Health Improvement Network database between 1990–2009. We estimated the incidence of fractures, burns and poisonings by age, sex, socio-economic group and calendar period and adjusted incidence rate ratios (IRR) comparing the least and most socio-economically deprived areas over time. Estimates of the UK annual burden of injuries and the excess burden attributable to deprivation were derived from incidence rates.

**Results:**

The cohort of 979,383 children experienced 20,804 fractures, 15,880 burns and 10,155 poisonings, equating to an incidence of 75.8/10,000 person-years (95% confidence interval 74.8–76.9) for fractures, 57.9 (57.0–58.9) for burns and 37.3 (35.6–38.0) for poisonings. Incidence rates decreased over time for burns and poisonings and increased for fractures (p<0.001 test for trend for each injury). They were significantly higher in more deprived households (IRR test for trend p<0.001 for each injury type) and these gradients persisted over time. We estimate that 865 fractures, 3,763 burns and 3,043 poisonings could be prevented each year in the UK if incidence rates could be reduced to those of the most affluent areas.

**Conclusions:**

The incidence of burns and poisonings declined between 1990 and 2009 but increased for fractures. Despite these changes, strong socio-economic inequalities persisted resulting in an estimated 9,000 additional medically-attended injuries per year in under-5s.

## Introduction

Childhood injury is a major cause of preventable ill-health, disability and death, [1]. In England and Wales it is the second most common cause of childhood death (age 1–4) after cancer, [2] and results in substantial long term disability, [1]. Injuries disproportionately affect more socio-economically deprived families, [3]–[14]. Globally this health inequality is striking, with more than 95% of all injury-related child deaths around the world occurring in low and middle-income countries, [1]. However, health inequalities in injury persist within high income countries also. For example, a study in England and Wales showed that the average annual death rate from injury in 2001 for children under age 15 was more than 13 times higher for children whose parents were classed as never having worked compared to children whose parents were in managerial or professional occupations, [3].

Since the UK government has made clear its intention to reduce health inequalities (as indicated in the Health and Social Care Act 2012, [15]) and the inclusion of injury-related admissions in young people has been included as a key performance indicator in the Public Health Outcomes Framework for England, [16], monitoring changes in injury rates over time and across socio-economic groups has never been more important. However, whilst the European Union recommend that ‘accidents and injuries’ are included in the European Statistical System of Eurostat, [17], in the UK there are no national surveillance systems monitoring all medically-attended injuries and we do not currently have any audit data that allow exploration by socio-economic group at a population level. Available data on hospital admissions for injury are routinely available for England and Wales, but by definition comprise only the most severe injuries and do not contain information on socio-economic status. Many more injuries are likely to be attended to in primary care, walk-in centres or emergency departments (EDs) that are not currently reported on at a population level in the UK. From 1978 to 2002 data on injury occurrence was captured from a sample of EDs by the Home and Leisure Accident Surveillance System (HASS & LASS) but these data are now more than 10 years out of date, are no longer collected and are not available via the interactive online portal.

Given the importance of injury in children in terms of the short and long term morbidity and mortality, we have used data from a nationally representative primary care database to estimate injury rates, the disease burden in terms of numbers of children injured and estimates of the number of excess injuries that are attributable to socio-economic deprivation.

## Methods

### Study design and data source

We conducted a cohort study using prospectively-collected health data from 495 General Practices from across the UK (i.e. England, Scotland, Wales and Northern Ireland) that contribute to The Health Improvement Network (THIN) research database. THIN includes all health information reported to primary care, including symptoms, diagnoses and treatments which are encoded into the medical record using Read codes where diagnoses are based on the International Classification of Diseases version 10 (ICD10). In the UK the general practitioner (primary care physician or family doctor) holds electronic health records for their patients and these records contain information about primary care consultations and importantly, other types of healthcare utilisation such as hospital admission into secondary and tertiary care, thus providing a comprehensive source of health information. Practices that contribute to THIN are broadly representative of all general practices in the UK in terms of age and sex of patients, practice size, geographical distribution and data quality, [18], are thought to be complete enough for research, [19] and acceptable outcomes from regular data quality checks and audits are a condition of participation in the network. In addition, primary care data have been shown to be a reliable source of medical information for a range of health issues, [20].

We used individual medical records from an open cohort of 979,383 children born between the 1^st^ of January 1990 and the 31^st^ of December 2009 who were registered with a THIN general practice before the age of 5 years. Children entered the cohort i.e. medical records were included from the latest date of when the practice started contributing data to THIN, when the practice had acceptable mortality data, the registration of the child with the practice or birth if the child was registered with the practice within 30 days of birth. Follow up ended at the earliest date of the most recent data collection from practices, when the child transferred out of the practice, died, or the day before their 5^th^ birthday. In the UK, records are automatically created when a new born baby is registered with a GP for the first time and records for existing patients are automatically transferred between practices if patients register with a new GP and are not reliant therefore on patients requesting that records are transferred. However we undertook a sensitivity analysis, restricting the analysis to participants with a minimum of 6 months person time in order to ensure that the incidence patterns we found were not biased by injured participants being more or less likely to register or transfer in or out of a practice and therefore contribute more or less person time to the study.

### Outcome; injury types

We studied the three most common types of medically attended injury incurred by children under the age of 5 internationally, [1]: fractures (any site or severity), burns (any site or severity and including scalds and flame burns) and poisonings (including those from medicinal and non-medicinal products/sources). Incident injuries in children’s records were defined by the presence of a diagnosis or treatment Read code in the medical record using comprehensive code lists for each injury type (available on request from authors). If a child had more than one injury code of the same type (e.g. Read codes for fracture at age 2 and again at age 4 years) both incident injuries were included. As some children had multiple Read code entries for the same injury type in close succession, we only considered new injury events as those with an interval of over 30 days between code entries to signify a new poisoning or burn event and over 100 days for a new fracture event. This was based on the analysis of Read code entry whereby intervals between children’s first and subsequent codes were plotted using a histogram and the point at which the curve levelled out was selected as the end of the event window. However, we also conducted a sensitivity analysis varying this window to over 90 days for poisonings, 90 days for burns and 300 days for fractures to determine the impact of potential overestimation of our initial event numbers.

### Exposure; socio-economic deprivation

Socio-economic deprivation was measured using the Townsend Index of material deprivation, in quintiles. This is an area-based composite score comprising measures of employment, car ownership, home ownership and overcrowding (i.e. number of adults per room) in an area of 400–600 households, [21]. Before general practices release their data to THIN, each patient is assigned a quintile of the Townsend index based on their home postcode and information from the 2001 UK census. This maintains patient anonymity and ensures that the patient’s quintile is representative of their relative socio-economic position at national level. The most recent home address at the time of data extraction is used to assign the index.

### Covariates

We assessed variation in injury by sex, age, calendar period, and socio-economic deprivation. Child age was divided into year intervals from birth, because of the known changes in risk of injury at different ages, [4], [22] and calendar time was divided into 5-year periods.

### Statistical methods

We calculated incidence rates (per 10,000 person years (PY)) and incidence rate ratios (IRR) using Poisson regression with a robust variance estimator for fractures, burns and poisonings. We mutually adjusted for sex, age, socio-economic deprivation and calendar period to provide adjusted incidence rate ratios (aIRR).

To assess whether deprivation and injury incidence rates varied over time we added terms for an interaction between deprivation quintile and calendar period to the models and tested statistical significance using a likelihood ratio test (LRT) with a *p* value smaller than 0.05 taken as statistically significant. In addition, we calculated adjusted injury IRRs between the most versus least socio-economically deprived groups at the start (1990–1994) and end (2005–2009) of the study period and conducted a test for trend (also using the LRT) for yearly incidence rate changes within Townsend quintiles.

To estimate the total burden of injuries in the UK in numbers we applied each year’s incidence rates (1990–2009) to the mid-year UK population estimates (1990–2009), [23], giving an annual number of injuries for each injury type and then summed these to produce a total estimate of injuries over the entire study and also for each 5-year study period. To assess how the burden had changed over time we subtracted the total number of injuries in the 1990–1994 period from the total number of injuries in the 2005–2009 period for each injury type. In addition, we estimated the excess incidence of injuries in the study population that could be attributed to deprivation using the population attributable risk calculation described by Steenland and Armstrong which takes account of different levels of deprivation exposure (i.e. the proportion of children in each quintile) [24]. In this calculation, the (IRR-1/IRR) is calculated for each of the top four quintiles of deprivation compared with the least deprived (bottom) quintile and this is then multiplied by the proportion of children in the study in that quintile of deprivation. These are then summed to give the final attributable risk fraction.

### Ethical approval

We used The Health Improvement Network (THIN) primary care database for this research. THIN data collection is undertaken by Cegedim Strategic Data and this has been approved by the UK South-East Multicentre Research Ethics Committee (SE-MREC). There is a standard process for managing ethical approval of individual studies that use these data which is managed by Cegedim's THIN Scientific Review Committee (SRC). A research protocol was submitted to the SRC and the protocol was approved in October 2009 (SRC Reference Number: 09–011). Patient informed consent is not required under this agreement nor is further additional ethics approvals from either the National Health Service ethics committees or from The University of Nottingham.

## Results

### Overall incidence rates

The study cohort comprised 979,383 children with a median study follow up time of 2.68 years (when they were under age 5). Fractures were the most common injury type; 20,804 fractures were incurred by 20,038 children (2% of the cohort), 15,880 burns were incurred by 15,286 children (1.5% of the cohort) and 10,155 poisonings were incurred by 9,772 children (1% of the cohort), all before the age of 5 (Table 1).

**Table 1:** Frequency of injury types in the study population (N = 979,383).

	Fractures	Burns	Poisonings
**Number of children with no injury**	959,472	964,176	969,611
**Number of children with at least one injury (% of all children)**	19,911 (2.03%)	15,207 (1.57%)	9,772 (1.00%)
**Total number of injuries**	20,668	15,796	10,155
**Number of injuries before age 5, per child (% of injured children)**			
1	19,206 (96.4%)	14,649 (96.3%)	9,417 (96.4%)
2	661 (3.3%)	528 (3.5%)	335 (3.4%)
≥3	44 (0.3%)	30 (0.2%)	20 (0.2%)

During the entire study period (1990–2009) the incidence of fractures was 75.8/10,000 PY (95% confidence interval 74.8–76.9), burn incidence was 57.9/10,000 PY (57.0–58.9) and poisoning incidence was 37.3/10,000 PY (36.5–38.0) (Table 2).

**Table 2:** Incidence rates of injury types by socio-demographic characteristics.

		Fractures		Burns		Poisonings	
	Person years(10,000years)	Frequency	Incidence rate(95% confidenceinterval) per 10,000person years	Frequency	Incidence rate(95% confidenceinterval) per 10,000person years	Frequency	Incidence rate(95% confidenceinterval) per 10,000person years
**Overall**	272.5	20,668	75.8 (74.8–76.9)	15,796	57.9 (57.0–58.7)	10,155	37.3 (36.5–38.0)
**Sex**							
Female	132.7	9,615	72.5 (71.0–73.9)	6,702	50.5 (49.3–51.7)	4,703	35.4 (34.4–36.5)
Male	139.9	11,053	79.0 (77.5–80.5)	9,094	65.0 (63.7–66.3)	5,452	39.0 (37.9–40.0)
**Age (years)**							
<1 year	53.2	1,488	27.9 (26.5–29.4)	2,989	56.1 (54.1–58.1)	710	13.3 (12.4–14.3)
1	56.7	4,175	73.6 (71.4–75.9)	6,486	114.4 (111.6–117.2)	3,184	56.2 (54.2–58.1)
2	55.6	4,847	87.2 (84.8–89.7)	3,284	59.1 (57.1–61.1)	3,605	64.9 (62.8–67.0)
3	54.2	4,884	90.0 (87.5–92.6)	1,810	33.4 (31.8–34.9)	1,821	33.6 (32.1–35.1)
4	52.8	5,274	99.9 (97.3–102.7)	1,227	23.2 (22.0–24.6)	835	15.8 (14.8–16.9)
**Socio-economic deprivation** **(Townsend Quintile)**							
1 (least deprived)	65.5	4,868	74.3 (72.2–76.4)	2,807	42.8 (41.3–44.7)	1,852	28.3(27.0–29.6)
2	52.2	3,901	74.7 (72.4–77.1)	2,461	47.1 (45.3–49.0)	1,766	33.8 (32.3–35.4)
3	51.6	3,879	75.1 (72.8–77.5)	2,996	58.0 (56.0–60.1)	2,035	39.4 (37.7–41.1)
4	48.5	3,783	78.0 (75.5–80.5)	3,436	70.8 (68.5–73.2)	2,180	44.9 (43.1–46.9)
5 (most deprived)	36.4	2,956	81.3 (78.4–84.2)	3,075	84.5 (81.6–87.6)	1,763	48.5 (46.2–50.8)
missing	18.3	1,281	70.0 (66.2–73.9)	1,021	55.8 (52.5–59.3)	559	30.5 (28.1–33.2)
**Calendar Period**							
1990–1994	21.2	1,431	67.4 (64.0–71.0)	1,914	90.2 (86.3–94.3)	1,177	55.5 (52.4–58.7)
1995–1999	62.3	4,701	75.4 (73.3–77.6)	3,740	60.0 (58.1–62.0)	2,779	44.6 (43.0–46.3)
2000–2004	89.4	6,621	74.0 (72.3–75.8)	5,061	56.6 (55.1–58.2)	3,198	35.8 (34.5–37.0)
2005–2009	99.6	7,915	79.4 (77.7–81.2)	5,081	51.0 (49.6–52.4)	3,001	30.1 (29.1–31.2)

### Incidence by age and sex

For each injury type boys experienced a higher rate of injury than girls (aIRR 1.09 (95% confidence interval 1.06–1.12) for fractures, 1.28 (1.24–1.33) for burns and 1.10 (1.06–1.14) for poisonings (Table 3)). Burns were most common at age 1 and poisonings at age 2, whereas fractures continued to increase with age (Tables 2 and 3).

**Table 3:** Unadjusted and adjusted incidence rate ratios for each injury type.

	Fractures		Burns		Poisonings	
	Incidence rate ratio(95% confidence interval)		Incidence rate ratio(95% confidence interval)		Incidence rate ratio(95% confidence interval)	
	Unadjusted	Adjusted+	Unadjusted	Adjusted+	Unadjusted	Adjusted+
**Sex**						
Female	1.00	1.00	1.00	1.00	1.00	1.00
Male	1.09 (1.06–1.12)	1.09 (1.06–0.12)	1.29 (1.25–1.33)	1.28 (0.24–0.33)	1.10 (1.06–1.14)	1.10 (1.06–1.14)
**Age (years)** *						
<1 year	1.00	1.00	1.00	1.00	1.00	1.00
1	2.64 (2.48–2.80)	2.64 (2.49–2.80)	2.04 (1.95–2.13)	2.06 (1.97–2.15)	4.21 (3.89–4.57)	4.25 (3.92–4.61)
2	3.12 (2.95–3.31)	3.13 (2.96–3.32)	1.05 (1.00–1.11)	1.07 (1.02–1.13)	4.87 (4.49–5.28)	4.97 (4.58–5.39)
3	3.22 (3.04–3.42)	3.24 (3.06–3.43)	0.59 (0.56–0.63)	0.61 (0.58–0.65)	2.52 (2.31–2.75)	2.60 (2.39–2.84)
4	3.58 (3.38–3.79)	3.60 (3.40–3.82)	0.41 (0.39–0.44)	0.43 (0.40–0.46)	1.19 (1.07–1.31)	1.25 (1.13–1.38)
**Socio-economic group** * **(Townsend Quintile)**						
1 (least)	1.00	1.00	1.00	1.00	1.00	1.00
2	1.01 (0.96–1.05)	1.01 (0.97–1.05)	1.10 (1.04–1.16)	1.10 (1.04–0.16)	1.20 (1.12–1.28)	1.20 (1.13–1.28)
3	1.01 (0.97–1.05)	1.02 (0.98–1.06)	1.35 (1.29–1.42)	1.35 (1.28–1.42)	1.39 (1.31–1.48)	1.40 (1.32–1.50)
4	1.05 (1.01–1.09)	1.06 (1.02–1.11)	1.65 (1.57–1.74)	1.64 (1.56–1.72)	1.59 (1.49–1.69)	1.60 (1.51–1.70)
5 (most)	1.10 (1.04–1.14)	1.11 (1.06–1.16)	1.97 (1.87–2.08)	1.94 (1.85–2.04)	1.71 (1.61–1.83)	1.72 (1.61–1.84)
missing	0.94 (0.88–1.00)	0.96 (0.90–1.02)	1.30 (1.21–1.40)	1.25 (1.17–1.35)	1.08 (0.98–1.19)	1.07 (0.97–1.17)
**Calendar Period** *						
1990–1994	1.00	1.00	1.00	1.00	1.00	1.00
1995–1999	1.12 (1.05–1.19)	0.95 (0.90–1.01)	0.66 (0.63–0.70)	0.81 (0.77–0.86)	0.80 (0.75–0.86)	0.85 (0.80–0.91)
2000–2004	1.10 (1.04–1.16)	0.93 (0.88–0.99)	0.63 (0.59–0.66)	0.77 (0.73–0.81)	0.64 (0.60–0.69)	0.69 (0.64–0.74)
2005–2009	1.18 (1.11–1.25)	1.01 (0.96–1.07)	0.56 (0.54–0.60)	0.67 (0.63–0.71)	0.54 (0.51–0.58)	0.57 (0.53–0.61)

*Likelihood ratio test for trend p<0.001 for fracture, burn and injury.

+ Mutually adjusted for sex, age, socio-economic deprivation and calendar period.

### Incidence by socio-economic deprivation

**Figure 1:**
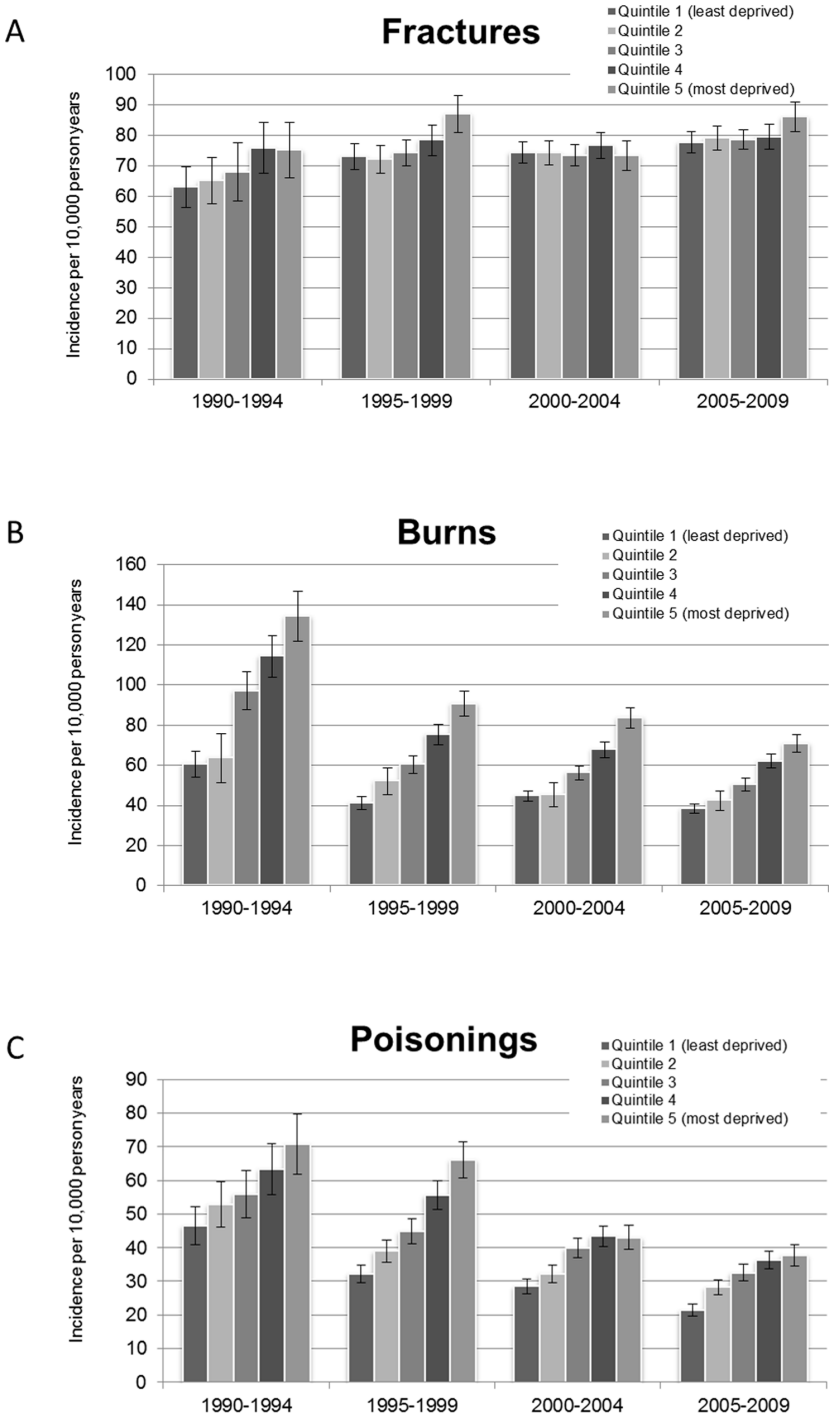
Incidence of fractures (A), burns (B) and poisonings (C) in 5-year periods. Columns represent each quintile of deprivation whereby 1 is the least deprived quintile and 5 is the most deprived quintile.

Rates for all injury types increased with increasing socio-economic deprivation (Table 2 and Figure 1, aIRR test for trend p<0.001 for each type). This gradient was steepest for burn injury with an absolute rate difference between children in the most versus the least deprived quintile of deprivation of 41.7/10,000 PY (aIRR comparing the most versus the least deprived quintile 1.94, 1.85–2.04). For poisoning there was a difference of 20.2/10,000 PY (aIRR 1.72, 1.61–1.84) and for fracture injury the absolute rate difference was only 7.0/10,000 PY (aIRR 1.11, 1.06–1.16). These gradients remained even when we restricted the analyses to participants with a minimum of 6 months person time.

However, the magnitude of this health inequality gap was not consistent over time within the study, particularly for burns and poisonings (test for interaction between calendar year and deprivation p = 0.29 for fracture, p = 0.01 for burn and p = 0.03 for poisoning) (Figure 1). For burns, in the period 1990–1994 there were 73.4/10,000 PY more injuries in the most deprived areas compared to the least (aIRR 2.22, 1.91–2.58) and this had fallen by 56% to 32.4/10,000 PY by the last study period, 2005–2009 (aIRR 1.79, 1.63–1.96). For poisonings, the absolute rate difference fell by 33% from 24.2/10,000 PY (adjusted IRR 1.51, 1.28–1.83) in 1990–1994 to 16.3/10,000 PY (adjusted IRR 1.75, 1.55–1.99) in 2005–2009 and for fractures fell by 32% from 12.1/10,000 PY (adjusted IRR 1.19, 1.00–1.41) to 8.24/10,000 PY (adjusted IRR1.13, 1.06–1.23).

### Incidence by calendar period

There was a statistically significant increase in fracture incidence rates over time (test for trend p<0.001) with an absolute rate increase of 12.0/10,000 PY from the earliest period (1990–1994) to the latest period (2005–2009). Conversely, burn and poisoning incidence rates significantly decreased over time (test for trend p<0.001 for both burn and poisonings) with an absolute rate reduction of 39.2/10,000 for burn injuries and 25.4/10,000 for poisoning injuries.

### Sensitivity analysis

When we used a more conservative approach to defining a new injury event by increasing the gap between Read code entries for the same injury type to over 90 days for poisonings, 90 days for burns and 300 days for fractures, the number of events reduced to 20,386 fractures, 15,658 burns and 10,103 poisonings. Therefore, this still captured the vast majority of events in our original analysis (97.9%, 98.6% and 99.9% of fractures, burns and poisonings respectively) indicating that most were likely to be true independent events. When we repeated all analyses of injury variation by age, sex, socio-economic status and calendar period our findings were almost identical to the original analyses.

### Annual UK estimates of medically attended fractures, burns and poisonings

Using UK national population estimates, [23], we calculated that there were 519,109 fractures, 471,519 burns and 284,606 poisoning events in under-5s across the UK during the study period. However, as described above, the incidence rates for fractures increased and for burns and poisonings decreased over the study period. Therefore we estimated that in the period 2005–2009 there were an additional 34,749 fractures, 79,251 fewer burns and 31,754 fewer poisonings when compared to the earliest study period (1990–1994).

### The population attributable risk

By calculating the attributable risk fraction, which assumes a causal relationship between deprivation and injury, we estimate that 3% of fractures, 30% of burns and 28% of poisonings could be attributed to deprivation. This means that per year 876 fractures, 5,485 burns and 3,034 poisonings could potentially be avoided if all children experienced the injury rates of those in the most affluent quintile of the population.

## Discussion

The incidence of burns and poisonings in young children has decreased substantially over the past 20 years, whereas the incidence of factures has increased to a smaller, but still important extent. However, despite these encouraging changes, important health inequalities have persisted over time, whereby children in deprived areas continue to experience significantly more injuries than children in more affluent areas. By calculating the population attributable risk we estimate that annually, there are an additional 9,395 medically-attended injuries in children under 5 that potentially, could be avoided if all children experienced the injury rates of those in the most affluent areas of the UK. It may be possible therefore to reduce injury rates even further by targeting interventions at those children in the most socio-economically deprived areas.

### Strengths and weaknesses of the study

As far as we are aware, this is the first study to estimate population-based incidence rates for the most common medically-attended childhood injuries that is not from self-reports of injury or a single source of presentation such as the emergency department or hospital admissions. Our cohort of nearly 1 million children is also the largest UK study to quantify injury-related health inequalities and how these have changed over time. Data show that THIN practice populations are broadly representative of the UK population in terms of demographics, disease prevalence and death rates, [18] and since in the UK approximately 98% of the population is registered with a GP [25] we believe that our study is generalizable to the wider UK population.

One potential explanation for our results is that participants from different socioeconomic backgrounds have different health seeking behaviour, resulting in injury ascertainment biases. For example, it is possible that people from lower socio-economic groups are more likely to register with a GP when an injury occurs, potentially inflating the incidence of injury in these groups. Whilst we cannot rule this out, only 0.4% of poisonings, 0.7% of burns and 0.8% of fractures were recorded within a week of registration with the GP practice. We did find that people from lower socio-economic groups contributed less person time to the study but when we restricted the analyses to people with at least 6 months of person time we found the same socio-economic gradients. This suggests that our results are not wholly explained by different patterns of health seeking behaviour from people in different socio-economic groups.

As with most studies using routinely-collected and medically-coded data, it is possible that some injuries have not been included because they were not Read-coded in the medical record. This may have led to underestimates of the crude incidence rates. However, there is little evidence to suggest that GP recording of injury using Read codes (rather than free text for example) is differentially influenced by a patient’s socio-economic status and GPs do not have access to patients’ Townsend scores which are added for research purposes when the data are downloaded from the practice.

We have estimated all incident injuries in children under the age of 5, however it is possible that we have misclassified new incident injuries as existing injuries and vice versa, under or overestimating the incidence rates. However the incidence rates we have presented are similar to those expected from other studies (see below) and our findings relating to health inequality gradients were robust when we undertook sensitivity analyses, changing the definition of new incident events. It is unlikely therefore that the health inequality patterns that we identified have been substantially affected by the way injury events have been defined.

A strength of this study was the use of an area-based measure of deprivation. Whilst this does not necessarily indicate an individual family’s economic wealth it does provide a more comprehensive measure of the environment that the family is exposed to which has been shown to be important [26]. We did not have access to individual-level socio-economic data however, it is likely that decisions to commission injury prevention efforts would be made at an area level (e.g. Clinical Commissioning Group area, local authority wards or districts) and our study would support decisions to fund prevention in the most deprived localities.

### Comparison with other studies

#### Injury incidence rates

Our data are consistent with previous studies. The incidence of fracture (75.8/10,000 person years across the 20 year study period) that we derived is comparable to data from European studies between 1988–2005 showing rates of fracture for children under five of between 50–100/10,000 person years,[27]–[29]. Likewise, our estimate of poisoning incidence (37.3/10,000 person years) is consistent with recent studies of ED attendances from high income countries such as Franklin et al who reported a poisoning incidence rate for 0–5 year olds in the USA in 2004 of 42.9/10,000, [30] and Xiang et al who reported a drug-poisoning incidence rate for 0–5 year olds of 25.5/10,000 in the USA, [31]. Few studies have described the incidence of burn injury in children at the population level in high income countries. Of the studies that have been published, most describe either fatalities or the incidence of severe burns that require inpatient admission whereas our study includes both primary and secondary care attended injuries. Our burn incidence rate of 57.9/10,000 person years is therefore much higher than such reports. For example, Vloemans et al reported an incidence of 16.3/10,000 admissions of 0–4 year olds to specialist burn centres in 2000–2007, [32] and Alaghehbandan et al reported a rate of 2.6/10,000 admissions for children aged 2–4 in Canada in 2012, [33].

#### Health inequalities

We detected a socio-economic gradient for fractures that differs from previous studies that have reported little or no association between fracture and deprivation, [4], [5], [34]. It may be that this is due to our very large study size and increased power to detect small differences between socio-economic groups. Descriptions of poisonings and burns being associated with increased deprivation are more consistent in the literature and our incidence rate ratios are similar to those reported previously. For example we reported a 72% increase in incidence of poisoning in the most compared to the least deprived areas, similar to the incidence rate ratio found by Xiang et al of 1.63 (drug-related poisonings for all ages), [31]. We found that across the study period, children in the most deprived areas were nearly twice as likely to have a burn injury compared to the most affluent areas. Other studies have shown a similar gradient. For example Hippisley-Cox et al showed that children under 15 in the most deprived areas in the East Midlands region of the UK were over three times more likely to have a hospital admission due to a burn or scald, [14] and Mulvaney et al showed that in 2004 for all ages, people in the most deprived quartile of areas were over 70% more likely to have a fire-related injury, [6].

### Public health implications

We have shown that despite large decreases in the incidence of burns and poisonings in young children since 1990, substantial inequalities persist between social groups in the UK. In addition, the incidence of fractures is increasing and smaller, yet important inequalities in fracture incidence exist. We estimate that up to 30% of burns and poisonings and 3% of fractures could be avoided if injury prevention interventions successfully reduced injury rates in the poorest areas to levels seen in the most affluent areas. This could result in an estimated 9,395 fewer medically-attended injuries per year across the UK.

A range of injury prevention interventions have been identified to reduce the types of health inequality that we have shown. The National Institute for Health and Care Excellence (NICE) recommends that safety assessments are undertaken in the most vulnerable households and that where appropriate, safety advice is given and equipment is provided and fitted by professionals to help prevent injury occurring in young children, [35]. In the US the ‘Protect the ones you love’ initiative has given rise to a multi-faceted national action plan for child injury prevention that includes elements of education, enforcement and environmental changes (so called 3E’s) that can be targeted at the highest risk neighbourhoods and families.

The reduction of inequalities in health, including injuries, is a matter of social justice and international organisations such as the WHO and UNICEF have shown their commitment to reducing these inequalities through the Parma declaration 2010, [36] and the World report on child injury prevention 2012, [1]. In England, Clinical Commissioning Groups and Local Authorities have responsibilities to reduce health inequalities and need to follow NICE guidance to achieve continued reductions in injury incidence and greater equity across social groups. Further to this, policy makers should include the reduction of injury-related health inequalities as an outcome measure in itself, rather than focusing on injury rates alone. In doing this, local health commissioners/decision makers are more likely to monitor and act upon health inequalities as recommended by NICE. An example of this is the Public Health Outcomes Framework in England which includes the reduction of injuries in young people as one of its indicators but this is measured by hospital admission rates, with no emphasis on the reduction of the health inequality gradient.

## References

[1] Peden M, Oyegbite K, Ozanne-Smith J, Hyder A, Branche C, et al.. (2008) World Report on child injury prevention. World health Organisation, Unicef. 26269872

[2] Statistics OfN (2012) Death registration summary tables - England and Wales, 2011 (final). Part of the Death Registrations summary tables.

[3] Edwards P, Roberts I, Green J, Lutchmun S (2006) Deaths from injury in children and employment status in family: analysis of trends in class specific death rates. BMJ 333: 119. 16829537 10.1136/bmj.38875.757488.4F PMC1502180

[4] Orton E, Kendrick D, West J, Tata LJ (2012) Independent risk factors for injury in pre-school children: three population-based nested case-control studies using routine primary care data. PLoS One 7: e35193. 22496906 10.1371/journal.pone.0035193 PMC3320631

[5] Lyons R, Delahunty A, McCabe M, Allen H, Nash P (2000) Incidence of childhood fractures in affluent and deprived areas: population based study. British Medical Journal 320: 149–149. 10634734 10.1136/bmj.320.7228.149 PMC27261

[6] Mulvaney C, Kendrick D, Towner E, Brussoni M, Hayes M, et al (2009) Fatal and non-fatal fire injuries in England 1995–2004: time trends and inequalities by age, sex and area deprivation. J Public Health (Oxf) 31: 154–161. 19074453 10.1093/pubmed/fdn103

[7] Kendrick D, Mulvaney C, Watson M (2009) Does targeting injury prevention towards families in disadvantaged areas reduce inequalities in safety practices? Health Educ Res 24: 32–41. 18203681 10.1093/her/cym083

[8] Roberts I, Power C (1996) Does the decline in child injury mortality vary by social class? A comparison of class specific mortality in 1981 and 1991. BMJ 313: 784–786. 8842070 10.1136/bmj.313.7060.784 PMC2352192

[9] Brown GW, Davidson S (1978) Social class, psychiatric disorder of mother, and accidents to children. Lancet 1: 378–381. 75409 10.1016/s0140-6736(78)91097-8

[10] Haynes R, Jones AP, Reading R, Daras K, Emond A (2008) Neighbourhood variations in child accidents and related child and maternal characteristics: does area definition make a difference? Health Place 14: 693–701. 18166497 10.1016/j.healthplace.2007.11.001

[11] Reading R, Jones A, Haynes R, Daras K, Emond A (2008) Individual factors explain neighbourhood variations in accidents to children under 5 years of age. Soc Sci Med 67: 915–927. 18573579 10.1016/j.socscimed.2008.05.018

[12] Kendrick D, Marsh P (2001) How useful are sociodemographic characteristics in identifying children at risk of unintentional injury? Public Health 115: 103–107. 11406774 10.1038/sj.ph.1900737

[13] Pomerantz WJ, Dowd MD, Buncher CR (2001) Relationship between socioeconomic factors and severe childhood injuries. J Urban Health 78: 141–151. 11368193 10.1093/jurban/78.1.141 PMC3456205

[14] Hippisley-Cox J, Groom L, Kendrick D, Coupland C, Webber E, et al (2002) Cross sectional survey of socioeconomic variations in severity and mechanism of childhood injuries in Trent 1992–7. BMJ 324: 1132. 12003886 10.1136/bmj.324.7346.1132 PMC107914

[15] Office. TS (2012) Health and Social Care Act 2012. In: Government U, editor. Chapter 7.

[16] (2012) Public Health Outcomes Framework. In: Health. Do, editor: Crown Copyright.

[17] Parliament E (2008) Regulation on Community statistics on public health and health and safety at work. In: Parliament E, editor. OJ L 354/70. Official Journal of the European Union.

[18] Blak BT, Thompson M, Dattani H, Bourke A (2011) Generalisability of The Health Improvement Network (THIN) database: demographics, chronic disease prevalence and mortality rates. Inform Prim Care 19: 251–255. 22828580 10.14236/jhi.v19i4.820

[19] Bourke A, Dattani H, Robinson M (2004) Feasibility study and methodology to create a quality-evaluated database of primary care data. Inform Prim Care 12: 171–177. 15606990 10.14236/jhi.v12i3.124

[20] Herrett EL, Thomas SL, Smeeth L (2011) Validity of diagnoses in the general practice research database. Br J Gen Pract 61: 438–439. 10.3399/bjgp11X583092 PMC3123481 21722461

[21] Townsend P, Phillimore P, Beattie A (1988) Health and deprivation: inequality and the North. London: Croom Helm.

[22] MacInnes K, Stone DH (2008) Stages of development and injury: an epidemiological survey of young children presenting to an emergency department. BMC Public Health 8: 120. 18410685 10.1186/1471-2458-8-120 PMC2330035

[23] Statistics. OfN All releases of Population Estimates for UK, England and Wales, Scotland and Northern Ireland.

[24] Steenland K, Armstrong B (2006) An overview of methods for calculating the burden of disease due to specific risk factors. Epidemiology 17: 512–519. 16804473 10.1097/01.ede.0000229155.05644.43

[25] Centre. HaSCI (2012) Attribution Data Set GP-Registered Populations 2010.

[26] Roberts I, Marshall R, Norton R, Borman B (1992) An area analysis of child injury morbidity in Auckland. Journal of Paediatrics and Child Health 28: 438–441. 1466939 10.1111/j.1440-1754.1992.tb02713.x

[27] Cooper C, Dennison EM, Leufkens HG, Bishop N, van Staa TP (2004) Epidemiology of childhood fractures in Britain: a study using the general practice research database. J Bone Miner Res 19: 1976–1981. 15537440 10.1359/JBMR.040902

[28] Mayranpaa MK, Makitie O, Kallio PE (2010) Decreasing incidence and changing pattern of childhood fractures: A population-based study.[Erratum appears in J Bone Miner Res. 2011 Feb;26(2): 439]. Journal of Bone & Mineral Research 25: 2752–2759. 20564246 10.1002/jbmr.155

[29] Rennie L, Court-Brown CM, Mok JY, Beattie TF (2007) The epidemiology of fractures in children. Injury 38: 913–922. 17628559 10.1016/j.injury.2007.01.036

[30] Franklin RL, Rodgers GB (2008) Unintentional Child Poisonings Treated in United States Hospital Emergency Departments: National Estimates of Incident Cases, Population-Based Poisoning Rates, and Product Involvement. Pediatrics 122: 1244–1251. 19047241 10.1542/peds.2007-3551

[31] Xiang Y, Zhao W, Xiang H, Smith GA (2012) ED visits for drug-related poisoning in the United States, 2007. Am J Emerg Med 30: 293–301. 21367556 10.1016/j.ajem.2010.11.031

[32] Vloemans AF, Dokter J, van Baar ME, Nijhuis I, Beerthuizen GI, et al (2011) Epidemiology of children admitted to the Dutch burn centres. Changes in referral influence admittance rates in burn centres. Burns 37: 1161–1167. 21726947 10.1016/j.burns.2011.05.001

[33] Alaghehbandan R, Sikdar KC, Gladney N, MacDonald D, Collins KD (2012) Epidemiology of severe burn among children in Newfoundland and Labrador, Canada. Burns 38: 136–140. 22103990 10.1016/j.burns.2011.06.010

[34] Stark AD, Bennet GC, Stone DH, Christi P (2002) Association between childhood fractures and poverty: population based study. British Medical Journal 324: 457. 11859047 10.1136/bmj.324.7335.457 PMC65666

[35] NICE (2010) Strategies to prevent unintentional injuries among under-15s.

[36] Region WHOE (2010) Declaration on Environment and Health. 5th Ministerial Conference on Environment and Health, Parma, Italy.

