# Correction: Persistence of Health Inequalities in Childhood Injury in the UK; A Population-Based Cohort Study of Children under 5

**DOI:** 10.1371/journal.pone.0119358

**Published:** 2015-03-23

**Authors:** The PLOS ONE Staff

The cohort of 979,383 children experienced 20,668 fractures and 15,796 burns. 876 fractures and 5,485 burns could be prevented each year in the UK if incidence rates could be reduced to those of the most affluent areas.

These values are incorrect in the first and last sentences under the subheading “Results” in the Abstract. The correct first sentence is: The cohort of 979,383 children experienced 20,668 fractures, 15,796 burns and 10,155 poisonings, equating to an incidence of 75.8/10,000 person-years (95% confidence interval 74.8–76.9) for fractures, 57.9 (57.0–58.9) for burns and 37.3 (35.6–38.0) for poisonings. The correct last sentence is: We estimate that 876 fractures, 5,485 burns and 3,043 poisonings could be prevented each year in the UK if incidence rates could be reduced to those of the most affluent areas.

Some of these values are also incorrect in the second sentence of the first paragraph under the subheading “Overall incidence rates” in the Results section. The correct sentence is: Fractures were the most common injury type; 20,668 fractures were incurred by 20,038 children (2% of the cohort), 15,796 burns were incurred by 15,286 children (1.5% of the cohort) and 10,155 poisonings were incurred by 9,772 children (1% of the cohort), all before the age of 5(Table 1)[1].

## References

[1] Orton E, Kendrick D, West J, Tata LJ (2014) Persistence of Health Inequalities in Childhood Injury in the UK; A Population-Based Cohort Study of Children under 5. PLoS ONE 9(10): e111631 doi: 10.1371/journal.pone.0111631 25347771 10.1371/journal.pone.0111631 PMC4210227

